# Case Report: Delayed diagnosis: a case of left main coronary artery spasm

**DOI:** 10.3389/fcvm.2025.1520516

**Published:** 2025-02-14

**Authors:** Yaxin Zhi, Wei Sun, Ziqiao Zhang, Demin Liu

**Affiliations:** Department of Cardiology, The Second Hospital of Hebei Medical University, Shijiazhuang, China

**Keywords:** left main coronary artery (LMCA) spasm, angina, coronary angiography (CAG), coronary artery bypass grafting (CABG), coronary artery spasm (CAS)

## Abstract

Left main coronary artery (LMCA) spasm is an exceedingly rare but potentially fatal condition. We present a case of severe stenosis of LMCA found by coronary angiography (CAG) due to recurrent chest pain, and subsequently received coronary artery bypass grafting (CABG). Nine years later, the patient was readmitted to the hospital because of precordial discomfort. During hospitalization, CAG was performed once again and showed no significant stenosis in the LMCA, leading to the diagnosis of LMCA spasm. This case emphasizes to interventional cardiologists the critical need to consider the possibility of LMCA spasm when diagnosing LMCA lesions. It highlights the importance of thorough and proactive pretreatment and comprehensive clinical judgment to minimize the risk of misdiagnosis.

## Introduction

Coronary artery spasm (CAS) is an abnormal contraction of the epicardial coronary arteries caused by various factors, which can occur in both normal vessels and areas of plaque stenosis, resulting in partial or complete vessel occlusion and can trigger a spectrum of severe cardiac events such as angina, myocardial infarction, heart failure, and malignant arrhythmias, and may even result in sudden death (1). Coronary artery spasm is not uncommon, especially right CAS (2), and it has attracted considerable attention from interventional cardiologists. However, left main coronary artery (LMCA) spasm is relatively rare, often underestimated, and potentially misdiagnosed. The LMCA originates from the left aortic sinus and typically bifurcates into the left anterior descending artery (LAD) and the left circumflex artery (LCX). The LAD supplies the anterior wall of the left ventricle and the interventricular septum, while the LCX supplies the lateral and posterior walls of the left ventricle (3). Understanding the anatomy of the LMCA and its branches is crucial for diagnosing and managing LMCA spasm, as it helps differentiate between spasm-induced stenosis and atherosclerotic disease. Therefore, timely and definitive diagnosis, along with the establishment of appropriate treatment plans for patients, remains a significant challenge. Here, we present a case of a patient who inadvertently underwent coronary artery bypass grafting surgery due to LMCA spasm. We hope that this case report will raise further awareness among interventional cardiologists about this rare but critical condition.

## Case report

**Figure 1:**
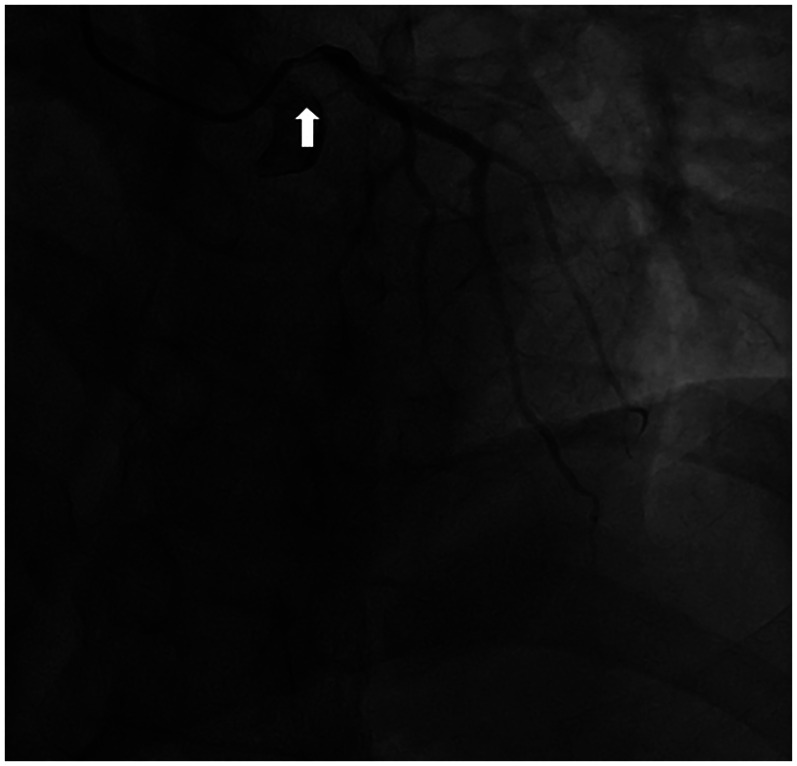
Coronary angiography was performed at the patient's first visit 11 years ago. A significant stenosis in the LMCA (arrow) was identified, suggesting the possibility of coronary artery spasm or atherosclerotic stenosis.

A 54-year-old man presented to the hospital 11 years prior due to recurrent chest pain. Coronary angiography (CAG) revealed stenosis of left main coronary artery (LMCA) and triple vessel disease (Figure 1). Considering the risk factors of coronary heart disease (type 2 diabetes and hyperlipidemia) and multi-vessel disease, the patient was referred to the Cardiac Surgery Department and underwent left internal mammary artery (LIMA) to left anterior descending artery (LAD) and aorta (AO) to diagonal branch (Diag) and posterior left ventricular branch (PLV). After discharge, the patient regularly underwent treatments for coronary dilation, reduction of myocardial oxygen consumption, lipid-lowering, and plaque stabilization.

**Figure 2:**
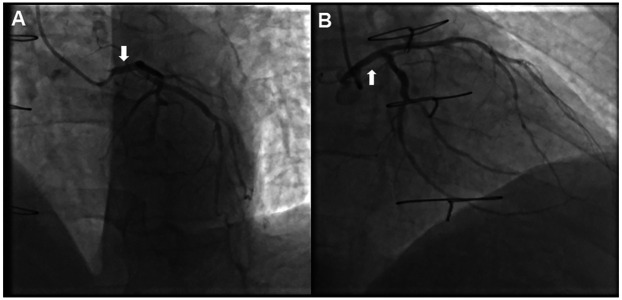
Coronary angiography was performed two years ago, showing the vascular conditions under different views. **(A)** No significant stenosis in the LMCA (arrow), compared to the previously stenosed area identified 11 years prior. **(B)** No significant stenosis in the LMCA (arrow), confirming the absence of organic narrowing and supporting the diagnosis of LMCA spasm.

Two years ago, the patient experienced recurrent precordial discomfort without any apparent cause. Coronary computed tomography angiography (CTA) revealed the following: (1) Changes after coronary artery bypass grafting: (a) Graft 1 originated from the left subclavian artery, with faint opacification in the proximal segment and no clear opacification in the mid to distal segments; (b) Graft 2 was mostly not opacified; (2) Coronary artery sclerosis; Atherosclerosis of the aorta. To further clarify the coronary artery lesions and the condition of the bypass grafts, the patient underwent CAG the next day, which revealed that the LMCA did not have significant stenosis, and at the same time, the bypass grafts had become occluded (Figures 2A,B). Therefore, we concluded that the stenosis of the left coronary artery found during the patient's first coronary angiography eleven years ago was due to spasm rather than plaque-induced narrowing. Upon this new diagnosis, the management strategy was revised to focus on medical therapy for potential LMCA spasm. The patient was prescribed a regimen of calcium channel blockers and nitrates to manage symptoms and prevent future spasmodic episodes. Additionally, lifestyle modifications were recommended, including smoking cessation and stress management, to address known risk factors for coronary artery spasm (CAS). Follow-up visits and periodic non-invasive cardiac imaging were scheduled to monitor the patient's condition and the effectiveness of the treatment. Over the subsequent six months, the patient reported a significant reduction in chest pain episodes, and follow-up assessments showed stable cardiac function without evidence of new coronary artery lesions. The patient's quality of life improved, and he remained free from severe cardiac events at the last follow-up, one year after the implementation of the new management plan.

## Discussion

Left main coronary artery (LMCA) spasm is a relatively rare but severe form of coronary artery spasm (CAS), which is infrequently reported in the literature (4). LMCA spasm manifests as symptoms of coronary heart disease and appears as localized stenosis of the LMCA in coronary angiography (CAG), which can easily lead to misdiagnosis as severe left main coronary atherosclerosis and result in inappropriate referral for surgical intervention, as seen in this case (5, 6). When LMCA spasm is suspected, initial treatment should focus on medical therapy rather than immediate surgical intervention. Medical treatment for CAS mainly includes calcium channel blockers and nitrates, which have been proven effective in relieving vasospasm and reducing ischemic symptoms (7, 8). In addition, lifestyle modifications such as smoking cessation and stress management are also important measures, as smoking is a significant risk factor for CAS and stress can trigger spasmodic episodes (7). This case underscores the critical importance of differentiating LMCA spasm from atherosclerotic disease to prevent unnecessary CABG and to implement appropriate medical therapy. It also highlights the potential long-term benefits of an accurate diagnosis and tailored management plan for LMCA spasm.

LMCA spasm can be either spontaneous or catheter-induced, and sometimes it is challenging to differentiate between the two. Hung et al. reported a case of a patient who developed LMCA stenosis during a treadmill exercise test, and the stenosis was alleviated upon the administration of an adequate dose of nitroglycerin during CAG, suggesting spontaneous LMCA spasm (9). Catheter-induced LMCA spasm, though rare, is a recognized complication of CAG (10, 11). Therefore, if left main stenosis is observed during this procedure, we must consider the possibility of catheter-induced vasospasm, which typically occurs within 1 mm of the catheter tip (12), and may result from mechanical irritation of the coronary artery wall by the catheter (13). Edris et al. (10) reported two cases of catheter-induced LMCA spasm. The first patient underwent CABG, and repeat CAG after 6 years showed a normal LMCA, while the second patient had a repeat angiogram just two days later showing a normal LMCA. In a large retrospective study of 7,295 coronary angiographies, Chang et al. (14) identified 30 cases of catheter-induced LMCA spasm (incidence rate of 0.41%). The use of various vasoconstrictive drugs is also a triggering factor for CAS. Hau et al. reported a case of a 42-year-old woman who suffered from severe CAS leading to acute myocardial infarction after taking a high dose of misoprostol for labor induction (15). Additionally, several literature reports have described the phenomenon of LMCA spasm induced by fluctuations in thyroid hormone levels (16–18). However, no abnormalities in thyroid function were found in our patient upon admission.

Previous cases have demonstrated that diagnosing LMCA spasm remains a challenge for interventional cardiologists. This process first necessitates a comprehensive assessment of clinical risk factors in patients, such as the presence of a smoking history, as smoking is the most significant risk factor for CAS. Additionally, it is recommended that thyroid function tests be included as a routine examination. When there is a high suspicion of vasospasm during CAG, such as isolated LMCA stenosis, which has an extremely low incidence (19), the intracoronary use of nitroglycerin is suggested as a standard practice, and provocation tests should be encouraged. Provocation tests with pharmacological agents (such as acetylcholine or ergonovine) during coronary angiography are considered the most reliable methods for diagnosing CAS (8, 20). The specific procedure involves the intracoronary injection of either acetylcholine or ergonovine, during which observations are made for any symptoms experienced by the patient, changes in electrocardiogram (ECG), and angiographic images that indicate CAS. A positive result is defined as transient, complete, or subtotal focal occlusion (>90% stenosis) of a coronary artery, accompanied by signs/symptoms of myocardial ischemia (angina and ischemic ECG changes), or induction of >90% diffuse vasoconstriction in two or more contiguous segments of a coronary artery (21). Furthermore, intravascular ultrasound (IVUS) and fractional flow reserve (FFR) can play crucial roles in differentiating coronary artery spasm from atherosclerotic disease (22–24). IVUS provides detailed images of the coronary artery lumen and wall, allowing for the identification of non-atherosclerotic causes of stenosis, such as vasospasm. FFR, on the other hand, measures the physiological significance of a coronary stenosis by assessing the ratio of distal coronary pressure to aortic pressure during maximal hyperemia. This functional assessment can help determine whether a stenosis is causing significant ischemia, thereby guiding appropriate therapeutic decisions (24).

## Conclusion

Inability to differentiate coronary artery spasm from left main coronary artery (LMCA) obstructive disease can lead to inappropriate referrals for coronary artery bypass grafting (CABG) surgery. The true incidence of unnecessary CABG in patients with LMCA spasm remains unknown. Therefore, it is imperative to enhance our capacity to identify LMCA spasm in order to prevent unnecessary revascularization procedures. In summary, a comprehensive approach that includes clinical assessment, advanced diagnostic tools (such as IVUS and FFR), and tailored medical therapy is essential for managing patients with suspected LMCA spasm.

## Data Availability

The original contributions presented in the study are included in the article/Supplementary Material, further inquiries can be directed to the corresponding author.
